# Phenolic Hydroxyl Groups in the Lignin Polymer Affect the Formation of Lignin Nanoparticles

**DOI:** 10.3390/nano11071790

**Published:** 2021-07-09

**Authors:** Jae Hoon Lee, Tae Min Kim, In-Gyu Choi, Joon Weon Choi

**Affiliations:** 1 Department of Agriculture, Forestry and Bioresources, Seoul National University, Seoul 08826, Korea; tirchonail@snu.ac.kr (J.H.L.); cingyu@snu.ac.kr (I.-G.C.); 2 Institute of Green-Bio Science and Technology, Seoul National University, Pyeongchang 25354, Korea; taemin21@snu.ac.kr; 3 Graduate School of International Agricultural Technology, Seoul National University, Pyeongchang 25354, Korea

**Keywords:** lignin, nanoparticle, fractionation, methylation, nanoprecipitation

## Abstract

Alkaline soda lignin (AL) was sequentially fractionated into six fractions of different molecular size by means of solvent extraction and their phenolic hydroxyl groups were chemoselectively methylated to determine their effect on nanoparticle formation of lignin polymers. The effect of the lignin structure on the physical properties of nanoparticles was also clarified in this study. Nanoparticles were obtained from neat alkaline soda lignin (ALNP), solvent-extracted fractions (FALNPs, i.d. 414–1214 nm), and methylated lignins (MALNPs, i.d. 516–721 nm) via the nanoprecipitation method. Specifically, the size properties of MALNPs showed a high negative correlation (*R*^2^ = 0.95) with the phenolic hydroxyl group amount. This indicates that the phenolic hydroxyl groups in lignin could be influenced on the nucleation or condensation during the nanoprecipitation process. Lignin nanoparticles exhibited high colloidal stability, and most of them also showed good in vitro cell viability. This study presents a possible way to control nanoparticle size by blocking specific functional groups and decreasing the interaction between hydroxyl groups of lignin.

## 1. Introduction

Lignin is a natural and three-dimensional phenolic polymer that accounts for 10–30% of the mass in lignocellulosic biomass. In recent decades, interest in the utilization of technical lignin as a high-value source has increased since around 70 million tons of lignin byproduct are generated annually worldwide in the pulping/paper industry (kraft, sulfite, etc.) and 10 million tons per year are generated in biorefineries [1,2]. However, 98% of the extracted lignin solution is just combusted for heat in pulp plants, and less than 2% of the lignin byproduct is converted into commercial products.

Formation of nanoparticles using lignin material is one of the promising applications to provide high value to it. Several studies have been carried out on lignin nanoparticle synthesis in a wide range of industrial fields. For example, lignin particles can be used as a reducing and capping agent to synthesize metallic nanoparticles [3]. Additionally, the synthesis of lignin/sodium dodecyl sulfate composite nanoparticles with high antiphotolysis and antioxidant properties was reported [4]. In addition, using lignin nanoparticles as materials for a novel drug delivery system may increase lignin value [5,6,7,8,9].

In previous studies, we evaluated the high potential of kraft lignin as a source of biocompatible nano-sized material and focused on the effect of the lignin structure on the sizes of the nanoparticles [10]. Six lignin fractions with significant differences in molecular size, weight, number of functional groups, internal linkages, and polydispersity were applied in the nanoprecipitation process. Correlations between particle sizes and several features of the lignin were determined. However, it was hard to clarify these correlations because of the considerable structural differences between each fraction and the polydispersity of the sizes of the nanoparticles. Therefore, further investigation of the effect of specific lignin structure is required.

The objective of this study was to examine and discuss the effect of hydroxyl group content in lignin on the physical properties of lignin nanoparticles, especially in terms of the particle size. The role of the phenolic hydroxyl group in determining the physicochemical properties of nanoparticles was investigated using functionalized lignin with different amounts of phenolic hydroxyl groups. First, an alkaline soda lignin (AL) from soda pulping was fractionated via sequential solvent extraction to obtain six different fractions with structural differences. At the same time, the phenolic hydroxyl group in AL was selectively blocked via methylation to eliminate the effect of the phenolic hydroxyl group on the lignin nanoprecipitation process. These phenolic groups were suspected of controlling the lignin nanoparticle growth via condensation. The formation of the AL-based nanoparticles was carried out using the nanoprecipitation method. The physicochemical properties (particle size, distribution, polydispersity, zeta potential, and colloidal stability) of the particles were determined using dynamic light scattering (DLS) and transmission electron microscopy (TEM). The differences in nanoparticle properties between phenolic hydroxyl group-blocked AL via methylation were carefully determined. The possible cytotoxicity was also assessed using the Cell Counting Kit-8 (CCK-8) test.

## 2. Materials and Methods

### 2.1. Materials

AL extracted from wheat straw was provided by Asian Lignin Manufacturing Pvt. Ltd., Chandigarh, India. Elemental analysis was performed with a 628 Series elemental analyzer sulfur add-on module (LECO Co., St. Joseph, MI, USA). Determination of structural carbohydrates, lignin, and ash was conducted, referred to the National Renewable Energy Laboratory (NREL) standard procedures [11,12]. The oxygen and carbohydrate contents were determined by difference (Table S1).

Acetone, 2-butanone, 1,4-dioxane, ethyl acetate, methanol, tetrahydrofuran (THF), dimethyl sulfate (DMS), dimethyl sulfoxide (DMSO), and dialysis tubing cellulose membranes (with *M*_w_ cut-off 14,000 Da) were purchased from Sigma-Aldrich Korea (Yongin, Korea).

### 2.2. Functionalization of Lignin

Fractionation of AL via sequential solvent extraction was performed according to our previous work [13]. AL was first dissolved in ethyl acetate for 2 h and vacuum filtered to separate dissolved liquid fractions and undissolved solid fractions. This fractionation process was repeated with 2-butanone, methanol, acetone, and a dioxane–water mixture (95:5 *v*/*v*). The recovered fractions were denoted FAL1, FAL2, FAL3, FAL4, FAL5, and FAL6 (1,4-dioxane-insoluble). The yield of each fraction was determined gravimetrically.

Chemoselective methylation of AL was conducted to block the phenolic hydroxyl group [14]. First, 1.6 g of AL was dissolved in 80 mL of aqueous 0.7 M sodium hydroxide at 25 °C. Dimethyl sulfate (0, 1, 2, and 6 mL) was then introduced to each AL and the mixture was heated to 70 °C for 2 h under vigorous stirring. The resulting products were then acidified with hydrochloric acid (pH < 2) to recover solid precipitates, followed by washing with deionized water three times and lyophilization. Those methylated lignins were denoted MAL0, MAL1, MAL2, and MAL6 depending on the amount of added dimethyl sulfate. The list of lignin samples used in this study are shown in Table 1.

### 2.3. Characterization of Lignin

Quantification of lignin hydroxyl group content in lignin was performed using ^31^P nuclear magnetic resonance (NMR) spectra [15]. Each lignin fraction dissolved in pyridine/CDCl_3_ mixture (1.6:1, *v*/*v*) and cyclohexanol as an internal standard was phosphitylated with 2-chloro-4,4,5,5-tetramethyl-1,3,2-dioxaphospholane (TMDP). These phosphitylated lignin samples were analyzed by an NMR instrument (AVANCE 600 MHz, Bruker, Billerica, MA, USA).

Lignin structures and lignin–carbohydrate complex linkages were quantified via 2D-^1^H-^13^C heteronuclear single quantum coherence (HSQC) NMR analysis (AVANCE 600 MHz, Bruker, Billerica, MA, USA) applying a pulse sequence “hsqcedetgpsp.3”, 32 scans, and acquisition of 1024 data points for ^1^H over 512 increments for ^13^C [16]. As a reference peak, central DMSO peak (δ_C_ = 40.1; δ_H_ = 2.5) was used. The MestReNova^®^ v12.0 software was employed to analyze HSQC spectra (Mestrelab Research, Santiago de Compostela, Spain) [17].

Methoxyl group content in lignin was determined using Baker’s method [18]. Lignin was reacted with hydroiodic acid at 130 °C for 30 min to release methyl iodide from the methoxyl groups, followed by the introduction of pentane and ethyl iodide (internal standard) under vigorous shaking. Finally, the pentane phase was analyzed using gas chromatography-mass spectrometry systems (5975C Series GC/MSD System, Agilent Technologies Inc., Santa Clara, CA, USA) to quantify the amount of methyl iodide formed by cleavage of the methoxyl group.

The number (*M*_n_) and weight average molecular weights (*M*_w_) of fractions were determined by a 1260 Infinity II LC System (Agilent Technologies Inc., Santa Clara, CA, USA) with a PLgel 5 μm MIXED-C column (300 mm × 7.5 mm, Agilent Technologies Inc., Santa Clara, CA, USA) for gel permeation chromatography (GPC). To obtain a molecular weight calibration curve, low molecular polystyrene standards (Mp 266–66,000 Da, PSS Polymer Standards Service GmbH, Mainz, Germany) were used.

### 2.4. Lignin Nanoparticle Formation

AL nanoparticle (ALNP) formation was conducted using a modified version of Lievonen’s method [19]. AL (1, 2, 4, and 6 mg) dissolved in THF (1.0 mL) was filtered with a 0.50 μm syringe filter and then introduced into dialysis tubing that was presoaked and washed. The tubing was immersed in 2 L of deionized water, which was exchanged at intervals of 3 h for over 12 h under 300 rpm stirring. The synthesized nanoparticles were denoted ALNP-C1, ALNP-C2, ALNP-C4, and ALNP-C6, respectively. Each experimental variable was run in triplicate.

Nanoparticles from AL fractions and methylated AL were then synthesized at a fixed concentration of 4 mg mL^−1^ THF based on the results in Section 3.2. Products from the fractions were denoted FALNP1, FALNP2, FALNP3, FALNP4, and FALNP5, respectively. Methylated lignin-derived particles were also denoted MALNP0, MALNP1, MALNP2, and MALNP6, respectively. Each variable was also run in triplicate.

### 2.5. Characterization of Lignin Nanoparticle

A transmission electron microscopy (TEM) image was obtained with the use of a LIBRA^®^ 120 (Carl Zeiss, Oberkochen, Germany). The samples were deposited on a thin carbon-coated copper 300 mesh TEM grid (Ted Pella, Inc., Redding, CA, USA).

Mean diameter, polydispersity (PDI, the square of standard deviation/the square of mean diameter), and the electrokinetic potential of ALNPs in a colloidal dispersion via DLS were calculated using a Zetasizer Nano ZS instrument (Malvern Panalytical Ltd., Worcestershire, UK) along with a polystyrene cuvette (peak and z-average size, DTS0012, Malvern Panalytical Ltd., Worcestershire, UK) and a folded capillary zeta cell (zeta potential, DTS1070, Malvern Panalytical Ltd., Worcestershire, UK). Each sample was diluted in deionized water and measured at 25 °C. The measured electrokinetic potential was converted into zeta potential using Smoluchowski’s formula [20]. Each sample was run in triplicate.

### 2.6. Cytotoxicity Test

In vitro cytotoxicity tests of ALNPs (C4, FALNP1, 2, and 3) on A549 cells (ATCC^®^, CCL-185^™^, Manassas, VA, USA) and Lewis lung carcinoma cells (LLC, ATCC^®^, CRL-1642^™^) were conducted using CCK-8 assays. Cell lines were grown in Dulbecco’s modified Eagle medium (DMEM, Thermo Fisher Scientific, Waltham, MA, USA) containing 10% fetal bovine serum (FBS, Atlas Biologicals, Fort Collins, CO, USA) and 1% antibiotic–antimycotic solution (ABAM, GeneDireX, Las Vegas City, NV, USA). Grown cells were seeded and attached to 96-well plates (3 × 10^3^ cells per well) overnight. After the medium was removed, serum-free DMEM with 1% ABAM and ALNPs with various concentrations (25, 50, 100, and 250 μg mL^−1^) were added and cultured for multiple time durations (24, 48, and 72 h for A549 and 8, 16, and 24 h for LLC). To examine cell viability, 10 μL of CCK-8 (Dojindo Molecular Technologies Inc., Kumamoto, Japan) was added into each well, followed by incubation for 3 h at 37 °C. Subsequently, the absorbance at 450 nm was measured using a microplate reader (Sunrise^™^, TECAN Group Ltd., Männedorf, Switzerland). Cells incubated with DMEM supplemented with 10% FBS and 10% Triton X-100 (TX, LPS solution, Daejeon, Republic of Korea) were used as positive and negative controls, respectively. Three replicates were used for each assay. All results were reported as the mean ± standard deviation (*n* = 3). Statistical differences among groups were analyzed using analysis of variance (ANOVA), and multiple t-tests were performed to compare differences between two groups. A *p*-value of <0.05 was considered significant.

## 3. Results and Discussion

### 3.1. Characteristics of Functionalized Lignin

#### 3.1.1. Lignin Fraction

AL was fractionated via sequential extraction with five different organic solvents, which were chosen based on several solvent properties such as the Hildebrand solubility parameter and Hansen solubility parameter. The solvency of selected solvents is given by a numerical value, the Hildebrand solubility parameter, which is an accurate representation of the square root of the cohesive energy density of the solvent. On the other hand, the Hansen solubility parameter utilizes the values of dispersion, polar, and hydrogen-bonding components of the Hildebrand parameter to quantify solvent–polymer compatibility. The parameters and properties of each solvent used in this study are presented in Table S2.

The yields of the six fractions (FAL1, 2, 3, 4, 5, and 6) were 8.9, 19.6, 32.5, 4.5, 15.4, and 19.1 wt%, respectively (Table 2 and Figure S1). The number average molecular weights (*M*_n_) and weight average molecular weights (*M*_w_) of the fractions were determined via GPC. AL had relatively small sizes and a uniform molecular structure with a *M*_w_ of 2880 Da and a dispersity (*M*_w_/*M*_n_) of 2.6 compared to the LignoBoost kraft lignin with 4580 Da and a dispersity of 3.1 [10]. After fractionation, each fraction had a lower *M*_w_/*M*_n_ compared to raw AL. The *M*_w_ values of the fractions increased from 1060 Da for FAL1 to 7790 Da for FAL5 as the fractionation progressed. However, GPC data from FAL6 could not be obtained because it did not dissolve in THF.

Quantitative analysis of functional group contents of the fractionated AL was performed (Table 3), and hydroxyl group regions of ^31^P NMR spectra are shown in Figure S2. As the sequential fractionation step progressed, total hydroxyl groups in the fractions decreased. Specifically, the amount of phenolic hydroxyl groups decreased from 2.84 mmol g^−1^ for FAL1 to 0.98 mmol g^−1^ for FAL5. In addition, the hydroxyl group from the diphenyl ether structure in FAL4 and 5 disappeared. Only phenolic hydroxyl group content in the syringyl unit did not show a clear decreasing trend (FAL2 > 1 > 4 > 3 > 5). Aliphatic hydroxyl group content increased during the fractionation process. However, the lowest aliphatic hydroxyl content was observed in FAL2, followed by 4 and 3, rather than in FAL1. No fractions showed aliphatic hydroxyl group content over raw AL. Thus, FAL6, the insoluble fraction, had a relatively higher free aliphatic hydroxyl group content.

^1^H-^13^C HSQC spectra were analyzed to determine the structure related to phenylpropanoid units and interunit linkages in the fractions. Quantitative measurement of main interunit linkages in AL, β-aryl ether (β-O-4), resinol (β-β), and phenylcoumaran (β-5), was performed using total aromatic unit spectra as internal standards [17]. Relative amounts of main interunit linkages in AL and derived fractions are listed in Table 4; the main signals in the sidechain and aromatic regions of NMR spectra are shown in Figures S3 and S4, respectively. In the raw AL spectra, signals of major linkages and aromatic groups were very weak, implying its very low abundance of cleavable β-O-4, and a highly condensed molecular structure. There was a strong signal (δ_C_/δ_H_ 75.4/3.5) that belonged to the phenylglycerol structure, which was likely generated from the cleaved nonphenolic β-O-4 ether bonds by NaOH [21]. The amounts of β-O-4, β-β, and β-5 in AL per 100 aromatic units were 5.8, 5.4, and 2.7, respectively. In the FAL1 and 2 spectra, strong cross signals of C–H from aromatic moieties were determined, but the signals of C–H from internal linkages were much weaker than the raw one. Therefore, it could be concluded that these light lignin fractions have higher phenolic hydroxyl group content and a highly condensed oligomer structure. FAL4 and 5 showed the highest amount of β-β and β-O-4 linkages, respectively, among the lignin fractions. Note that the cross signals of proton and carbon from FAL4 and 5 were, unfortunately, too weak to predict their molecular structure.

#### 3.1.2. Methylated Lignin

Phenolic hydroxyl group-selective methylation was carried out in neat DMS as a solvent at 70 °C for 2 h using NaOH due to its relatively mild condition. Quantification of lignin hydroxyl group content in each methylated lignin was performed using ^31^P NMR spectra. The number and weight average molecular weights of a series of methylated lignins were also determined via GPC.

Quantitative determination of the phenolic hydroxyl group showed that AL was well methylated (Table S3). After methylation, total phenolic hydroxyl groups significantly decreased with an increased amount of reacted DMS, from 2.15 mmol g^−1^ for AL to 0.17 mmol g^−1^ for MAL6, while carboxylic acid also decreased from 1.31 mmol g^−1^ for AL to 0.15 mmol g^−1^ for MAL6. However, the amount of aliphatic hydroxyl groups slightly decreased from 2.38 mmol g^−1^ for AL to 2.35 mmol g^−1^ for MAL2 and 1.73 mmol g^−1^ for MAL6. This indicates that specific methylation of AL was successfully performed.

The *M*_n_ and *M*_w_ determined via GPC shows that the difference between the *M*_w_ value of AL and MAL0 (heated but non-methylated) was not significant (Table S3 and Figure S5). The *M*_w_ of methylated lignins increased from 3291 Da for MAL1 to 4243 Da for MAL6 as the degree of methylation increased. In addition, *M*_w_/*M*_n_ of methylated lignin ranged from 2.4 to 3.2, which was not remarkably different from neat AL (2.6).

### 3.2. Effect of Lignin Characteristics on Nanoparticle Size

**Figure 1:**
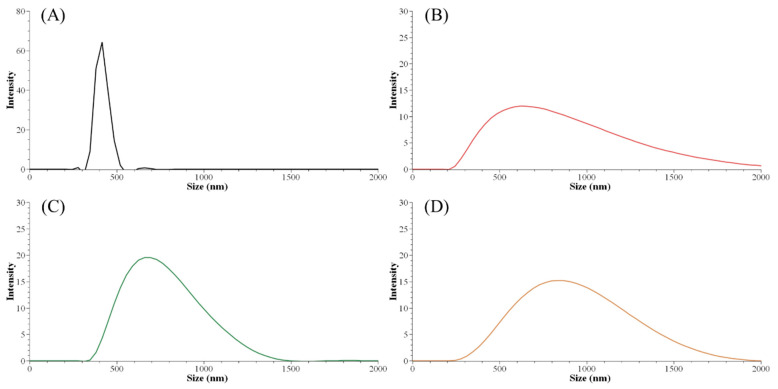
Particle size distribution graph of ALNPs with different initial concentrations. (**A**) ALNP-C1, (**B**) ALNP-C2, (**C**) ALNP-C4, and (**D**) ALNP-C6.

The initial lignin concentration before the precipitation process guided the nanoparticle size distribution. The smallest Z-average size (harmonic intensity averaged size) of 671.9 nm and also the narrowest PDI among ALNP-Cs were obtained at 4 mg mL^−1^ (Table 5). By contrast, the smallest peak size of 414 nm was obtained at 1 mg mL^−1^ (Figure 1). However, ALNP-C1 presented the largest Z-average size among all samples (1903 nm) and a PDI value of 1.0, which is theoretically the highest value. Despite identifying a clear single peak in the graph, it is hard to dismiss the exceptional result of the Z-average cumulant analysis of the measured correlation curve of ALNP-C1 because this could happen when the largest peak is larger than the large cut-off of DLS (e.g., very large aggregates or dust). Therefore, ALNP-C4, which showed the smallest Z-average size, PDI, and the second-smallest peak size, were chosen to be representative nanoparticles in this study. On the other hand, the peak sizes and their standard deviation both increased as the lignin concentration increased (*R*^2^ = 0.66). But the Z-average sizes and PDI showed a weak decreasing trend as the predialysis concentration increased due to the outlier peak size from ALNP-C1 (*R*^2^ = 0.29).

**Figure 2:**
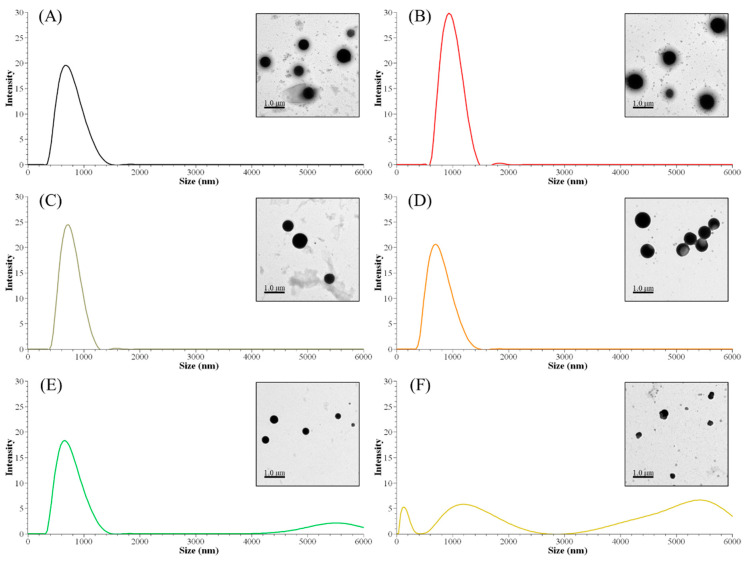
Peak size distributions of FALNPs and their representative TEM images. (**A**) ALNP-C4, (**B**) FALNP1, (**C**) FALNP2, (**D**) FALNP3, (**E**) FALNP4, and (**F**) FALNP5.

The nanoparticle size distribution was affected by the sequential solvent extraction. As shown in Table 4, particles derived from low-molecular-weight fractions had larger sizes than the control group (ALNP-C4) or high-molecular-weight groups. The largest nanoparticles consisted of FAL1 with lower *M*_w_ and higher hydroxyl group content. However, nanoparticles with similar sizes compared to the control were produced from fractions that had higher *M*_w_ and fewer hydroxyl groups (Figure 2). Since THF is exchanged to water during dialysis, the hydroxyl group and/or carboxylic group in the polymer could interact with water molecules. Thus, interfacial tension increases [22], nucleation rate decreases [23], and fewer initial nuclei form [24]. Because of the relatively small number of nuclei, each nucleus could grow larger until a solute concentration reaches below the equilibrium saturation concentration. In addition, the high tendency for self-association due to the strongly interacting surface hydroxyl groups in the fractions is considerable. Meanwhile, FALNP4 and 5 had multipeak size distributions. Three particular peaks of FALNP5 had very low intensity, which hints at varied size and unstable colloidal properties. 

There were no strong correlations between particle size and molecular characteristics of AL fractions. Specifically, fractions with higher *M*_w_ tended to form smaller nanoparticles (*R*^2^ = 0.77). On the other hand, the amounts of carboxylic, phenolic hydroxyl, and total hydroxyl groups were positively correlated with Z-average size of FALNPs (*R*^2^ = 0.67, 0.63, and 0.61, respectively). A negative correlation between the relative amount of β-O-4 and particle size was also significant (*R*^2^ = 0.68). Therefore, it could be assumed that the hydrogen bond formation between hydroxyl groups encourages nucleation or condensation during the nanoprecipitation process. However, the aliphatic hydroxyl group had a low correlation with particle size (*R*^2^ = 0.25). The morphologies of ALNPs were determined using TEM images. The particles had a spherical shape while FALNP5 showed relatively irregular spherical structures.

**Figure 3:**
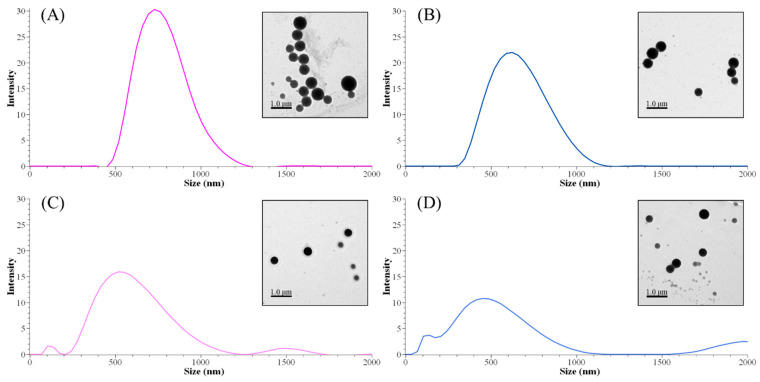
Peak size distributions of MALNPs and their representative TEM images. (**A**) MALNP0, (**B**) MALNP1, (**C**) MALNP2, and (**D**) MALNP6.

The methylation process affected the size distribution of lignin nanoparticles. Both peak sizes and Z-average sizes decreased by decreasing the content of the phenolic hydroxyl group and/or molecular weight (Table 6). Although there was no identified remarkable difference between neat AL and MAL0 structures, the Z-average size difference was significant (672 and 915 nm, respectively). However, the difference in a single peak of size between ALNP-C4 and MALNP0 was not meaningful. As shown in Figure 3, larger nanoparticles consisted of less-methylated lignin with a lower *M*_w_ and less-decreased phenolic hydroxyl group (MALNP0 and 1) content. Simultaneously, particles with a smaller but multipeak size distribution were obtained from highly methylated lignin with a higher *M*_w_ and largely decreased phenolic hydroxyl group content (MALNP2 and especially 6). This result is in agreement with previous works on the formation of nanoparticles from lignin fractions that have low hydroxyl group content. It is assumed that the higher phenolic hydroxyl group content is related to the synthesis of even-sized lignin nanoparticles.

There was a very high correlation between particle size and phenolic hydroxyl group content of methylated lignins (*R*^2^ = 0.95). Total hydroxyl group and carboxylic acid content also showed a very high or significant correlation with the particle size (*R*^2^ = 0.93 and 0.71, respectively). However, the effect of total hydroxyl group content does not seem meaningful due to the small change in aliphatic hydroxyl group content during the methylation. Consequently, it could be concluded that the phenolic hydroxyl group content in lignin is an important factor in nucleation or condensation during the nanoprecipitation process.

### 3.3. Particle Surface Charge

The zeta potential can be applied to determine electrokinetic potential in colloidal systems [25]. Zeta potential values are typically in the range of 100 to −100 mV, but nanoparticles with values >30 or <−30 could be considered to have a high degree of stability [26]. Lower dispersion zeta potential values promote van der Waals interparticle attraction and lead to aggregation, coagulation, or flocculation of nanoparticles [26,27].

**Figure 4:**
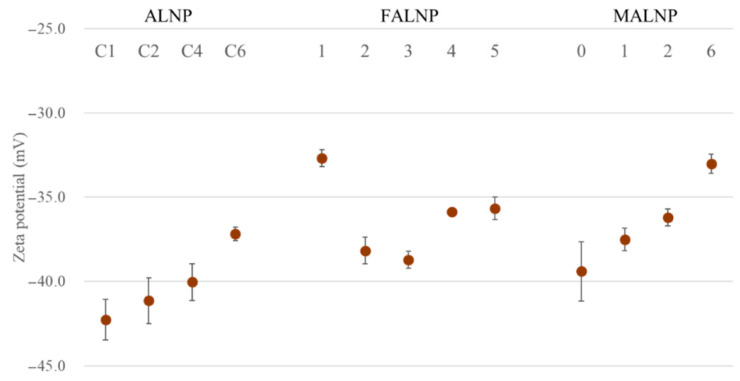
Zeta potential values of ALNPs as functions of various conditions.

Zeta potential values for all the ALNPs in this study exceeded −30 mV, which indicates relatively high-water stability. Predialysis concentration of lignin negatively affected the zeta potential (*R*^2^ = 0.96). The zeta potential value decreased from −42.3 mV for ALNP-C1 to −37.2 mV for ALNP-C6 (Figure 4). The methylation level of lignin hydroxyl groups also negatively affected zeta potential from −42.3 mV for MALNP0 to −33.0 mV for MALNP6, but the correlation (*R*^2^ = 0.69) was lower than that of the predialysis concentration.

In contrast, no significant relationships between zeta potential value and the solvent extraction process or the size of FALNP were observed. Moreover, FALNP1 showed the lowest colloidal dispersion stability among the samples, although FAL1 has the highest content of hydroxyl groups and carboxylic acids, which can give a negatively charged surface. Since each lignin fraction had a totally different molecular weight and other characteristics, we assume that the fractions showed different condensation characteristics with their functional groups during the nanoprecipitation process.

### 3.4. In Vitro Cell Viability

**Figure 5:**
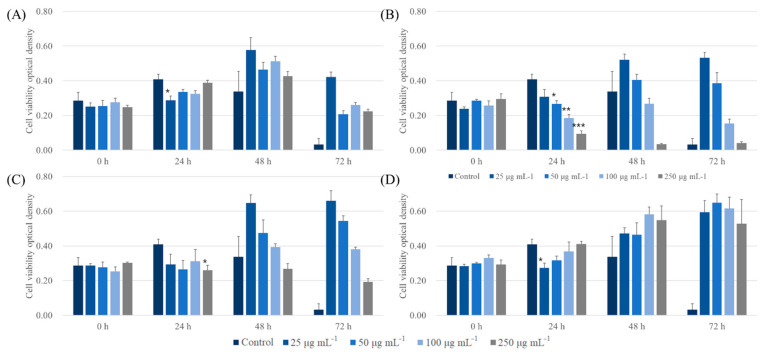
Cytotoxic effect of ALNPs on A549 cells determined by the Cell Counting Kit-8 assay. (**A**) ALNP-C4, (**B**) FALNP1, (**C**) FALNP2, and (**D**) FALNP3. All data sets were compared to the blank control (serum-free DMEM containing 1% of antibiotic–antimycotic solution). The levels of significant differences were set at probabilities of * *p* < 0.05, ** *p* < 0.01, and *** *p* < 0.001.

To determine the biocompatible potential of ALNPs, a CCK-8 assay was carried out on A549 and LLC cell lines. First, ALNPs exhibited a relatively high cell viability to A549 cell lines at 25 and 50 μg mL^−1^ (Figure 5). Specifically, ALNP-C4 showed no significant cytotoxicity in all concentrations that were tested. However, KLNP-F1 represented a decreasing trend of cell viability as a function of concentration. At 250 μg mL^−1^, a severe decrease of cell viability within 24 h and was observed, and the cell line was nearly completely dead within 72 h. On the other hand, the antiproliferation effect of FALNP2 and 3 was insignificant. Besides, the A549 cell line culture flourished more in the presence of FALNP2 and 3 compared to ALNP-C4.

**Figure 6:**
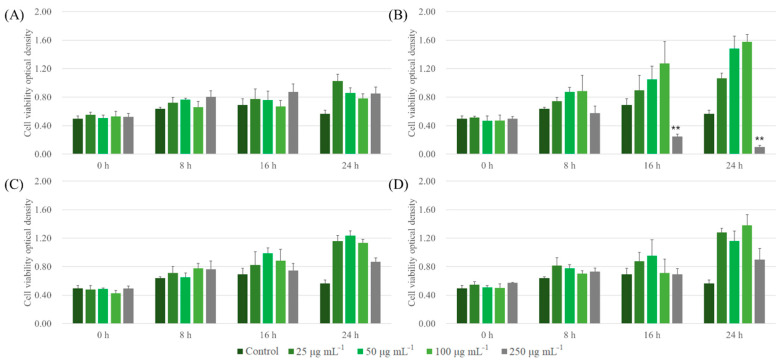
Cytotoxic effect of ALNPs on LLC cells determined by the Cell Counting Kit-8 assay. (**A**) ALNP-C4, (**B**) FALNP1, (**C**) FALNP2, and (**D**) FALNP3. All data sets were compared to the blank control (serum-free DMEM containing 1% of antibiotic–antimycotic solution). The levels of significant differences were set at probabilities of * *p* < 0.05, ** *p* < 0.01, and *** *p* < 0.001.

Similar trends of cell cytotoxicity in the case of LLC were observed. ALNPs, except for FALNP1 at higher concentrations (100 and 250 μg mL^−1^), showed extremely low cytotoxicity to LLC cell lines at all concentrations tested (Figure 6). FALNP2 and 3 had no cytotoxic effect even at high concentrations. The cell proliferation effect of FALNP2 and 3 was lower but comparable to the positive control (data not shown). Therefore, we showed the high cell viability of ALNPs and the potential of AL as a source for drug delivery systems. Still, in vivo assays are needed to define the biocompatibility of lignin nanoparticles.

## 4. Conclusions

Sequential solvent extraction and chemoselective methylation of AL were carried out to block its phenolic hydroxyl groups and obtain fractions with different chemical properties or decrease the amount of specific functional groups. These modified lignin samples were then used to synthesize nano-sized spherical particles. A light weighted lignin fraction contained higher amounts of total functional groups and condensed structures. Additionally, FALNPs from the low-molecular-weight AL fractions had larger sizes. Methylation of lignin phenolic hydroxyl groups followed by synthesis of MALNPs clarified a high correlation between the phenolic hydroxyl group and average size of the nanoparticles. Every particle showed comparable and good colloidal stability, while there was a large size difference between each ALNP. In vitro cell viability tests showed that ALNPs had very low cytotoxicity (except FALNP1) at high colloidal concentrations, which encourages the potential use of ALNPs as a drug delivery system.

Since it was determined that lignin nanoparticle size could be controlled by blocking specific functional groups and decreasing the interaction between hydroxyl groups, further applications of lignin-based nanoparticles with ideal sizes is expected. Particle size-reduction for drug-encapsulated nanoparticles and increasing the size would be appropriate for other industrial uses such as UV blockers, anode materials, absorbents, or biocidal materials.

## Figures and Tables

**Table 1:** List of functionalized alkaline lignin samples.

Samples	Abbreviation	Conditions	Abbreviation
Alkaline soda lignin	AL		
Fractionated alkaline soda lignins	FAL	Ethyl acetate-fractionated	FAL1
		2-Butanone-fractionated	FAL2
		Methanol-fractionated	FAL3
		Acetone-fractionated	FAL4
		Dioxane–water mixture-fractionated	FAL5
		Dioxane–water mixture-insoluble	FAL6
Methylated alkaline soda lignins	MAL	Added 0 mg of dimethyl sulfate (DMS)	MAL0
		Added 1 mg of DMS	MAL1
		Added 2 mg of DMS	MAL2
		Added 6 mg of DMS	MAL6

**Table 2:** The yields of alkaline soda lignin fractions and their GPC information.

Samples	Yield (%)	*M*_w_	*M*_n_	*M*_w_/*M*_n_
AL		2880	1130	2.6
FAL1	8.9	1060	610	1.7
FAL2	19.6	1780	1050	1.7
FAL3	32.5	2920	1480	2.0
FAL4	4.5	4990	2420	2.0
FAL5	15.4	7790	1950	4.0
FAL6	19.1	ND ^*^	ND	ND

* Not dissolved in THF.

**Table 3:** Content in hydroxyl groups of fractionated AL quantified by ^31^P NMR and methoxy group.

Samples	Hydroxyl (mmol g^−1^)						Methoxy (mmol g^−1^)
	Phenolic				Carboxylic Acids	Aliphatic	
	H ^a^	G ^b^	S ^c^	4-O-5			
AL	0.29	0.91	0.81	0.14	1.31	2.38	3.59
FAL1	0.41	1.12	0.76	0.55	2.05	1.57	3.92
FAL2	0.38	1.11	0.91	0.09	1.41	1.10	3.97
FAL3	0.22	0.75	0.58	0.03	1.12	1.53	3.55
FAL4	0.14	0.60	0.69	ND ^d^	0.67	1.52	3.40
FAL5	0.10	0.46	0.42	ND	0.60	2.01	3.07

^a^ *p*-hydroxyphenyl unit; ^b^ guaiacyl unit; ^c^ syringyl unit; ^d^ not detected.

**Table 4:** Relative amounts of main interunit linkages in AL and its fractions.

Samples	Linkage Amount (per 100 Aromatic Units)			
	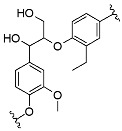	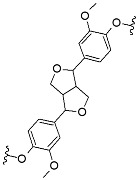	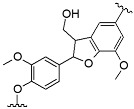	
	β-O-4	β-β	β-5	H:G:S
AL	5.8	5.4	2.7	32:48:20
FAL1	0.5	2.8	0.4	33:44:23
FAL2	1.9	1.5	0.4	35:48:17
FAL3	6.1	1.3	0.9	36:50:14
FAL4	8.8	9.0	ND	37:45:18
FAL5	12.1	ND	ND	4:82:14

ND: not detected.

**Table 5:** Size properties of fractionated AL-derived nanoparticles.

		Peak Size (nm)						Z-Average Size (nm)	PDI
		Peak 1	Intensity (%)	Peak 2	Intensity (%)	Peak 3	Intensity (%)		
AL nanoparticles (ALNPs) with different initial concentrations	C1	414	100	-	-	-	-	1903	1.000
	C2	741	100	-	-	-	-	729.6	0.346
	C4	721	100	-	-	-	-	671.9	0.118
	C6	840	100	-	-	-	-	959.9	0.381
Fractionated AL nanoparticles	FALNP1	953	100	-	-	-	-	1103	0.276
	FALNP2	732	100	-	-	-	-	704.6	0.002
	FALNP3	733	100	-	-	-	-	796.8	0.253
	FALNP4	701	97.0	5313	3.0	-	-	740.9	0.244
	FALNP5	128	50.5	1241	34.9	4942	14.5	286.9	0.731

**Table 6:** Size properties of methylated AL-derived nanoparticles.

	Peak Size (nm)						Z-Average Size (nm)	PDI
	Peak 1	Intensity (%)	Peak 2	Intensity (%)	Peak 3	Intensity (%)		
MALNP0	754	100	-	-	-	-	915.2	1.000
MALNP1	629	100	-	-	-	-	825.5	0.506
MALNP2	519	92.8	138	6.0	1480	1.1	553.5	0.390
MALNP6	586	74.6	87	22.2	1931	3.1	462.6	0.406

## Data Availability

The data presented in this study are available in the article and supplementary material.

## Supplementary Materials

The following are available online at https://www.mdpi.com/article/10.3390/nano11071790/s1. Figure S1. GPC curves of AL and its fractions by solvent extraction. Figure S2. 31P NMR spectra of (A) AL, (B) FAL1, (C) FAL2, (D) FAL3, (E) FAL4, and (F) FAL5. Figure S3. Sidechain regions in the 2D-HSQC NMR spectra of (A) AL, (B) FAL1, (C) FAL2, (D) FAL3, (E) FAL4, and (F) FAL5; Aα, Cα-Hα in β-O-4; Bα, Cα-Hα in β-β; Cα, Cα-Hα in phenylcoumaran; OMe, C-H in methoxyls; X, C-H in phenylglycerol. Figure S4. Sidechain regions in the 2D-HSQC NMR spectra of (A) AL, (B) FAL1, (C) FAL2, (D) FAL3, (E) FAL4, and (F) FAL5; Sn, Cn–Hn in syringyl units; Gn, Cn–Hn in guaiacyl units; Hn, Cn–Hn in hydroxyphenyl units. Figure S5. GPC curves of raw and methylated Als. Table S1. Chemical and thermal properties of wheat straw soda lignin (AL). Table S2. Solubility parameters and related properties of five different solvents used in this study. Table S3. Changing the content of phenolic hydroxyl groups in methylated AL quantified by 31P NMR and their GPC information.
