# Supplementary material for: Phenolic Hydroxyl Groups in the Lignin Polymer Affect the Formation of Lignin Nanoparticles
Source: Nanomaterials (Basel). 2021 Jul 9;11(7):1790. doi: 10.3390/nano11071790 (PMC8308409; doi:10.3390/nano11071790)
Supplement: Supplementary file 1 [file nanomaterials-11-01790-s001.zip › nanomaterials-1255058-supplementary.pdf]

Supplementary Materials

Article

Phenolic Hydroxyl Groups in the Lignin Polymer Affect the Formation of Lignin Nanoparticles

Jae Hoon Lee [1], Tae Min Kim [2,3], In-Gyu Choi [1,2] and Joon Weon Choi [2,3,*]

[1] Department of Agriculture, Forestry and Bioresources, Seoul National University, Seoul 08826, Korea;
tirchonail@snu.ac.kr (J.H.L.); cingyu@snu.ac.kr (I.-G.C.)

[2] Institute of Green-Bio Science and Technology, Seoul National University, Pyeongchang 25354, Korea;
taemin21@snu.ac.kr

[3] Graduate School of International Agricultural Technology, Seoul National University, Pyeongchang 25354, Korea

* Correspondence: cjw@snu.ac.kr; Tel.: +82-3-3339-5840; Fax: +82-3-3339-5689

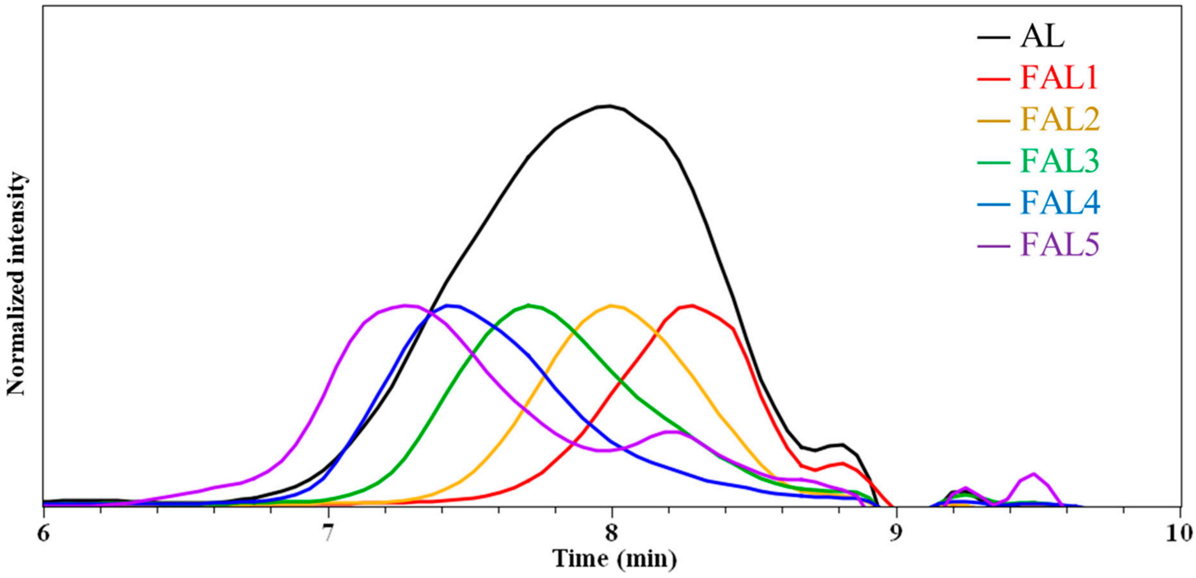

Figure S1. GPC curves of AL and its fractions by solvent extraction

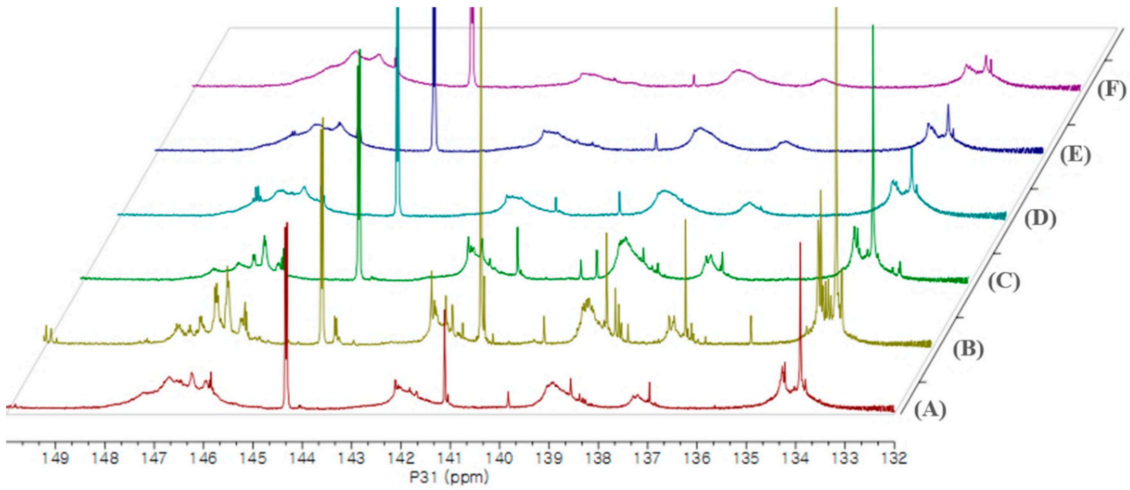

Figure S2. ^{31}P NMR spectra of (A) AL, (B) FAL1, (C) FAL2, (D) FAL3, (E) FAL4, and (F) FAL5

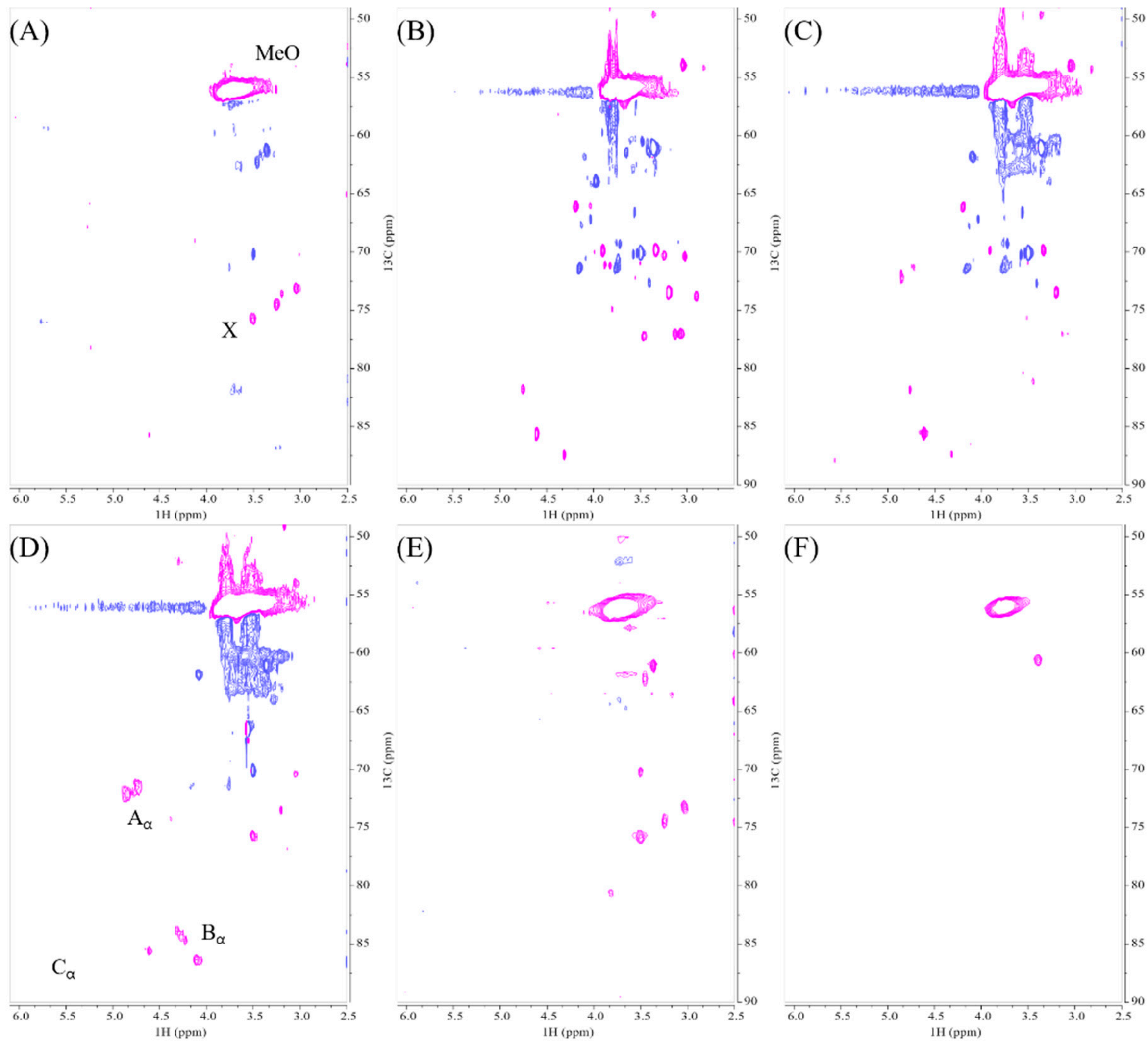

Figure S3. Sidechain regions in the 2D-HSQC NMR spectra of (A) AL, (B) FAL1, (C) FAL2, (D) FAL3, (E) FAL4, and (F) FAL5; Aα, Cα-Hα in β-O-4; Bα, Cα-Hα in β-β; Cα, Cα-Hα in phenylcoumaran; OMe, C-H in methoxyls; X, C-H in phenylglycerol.

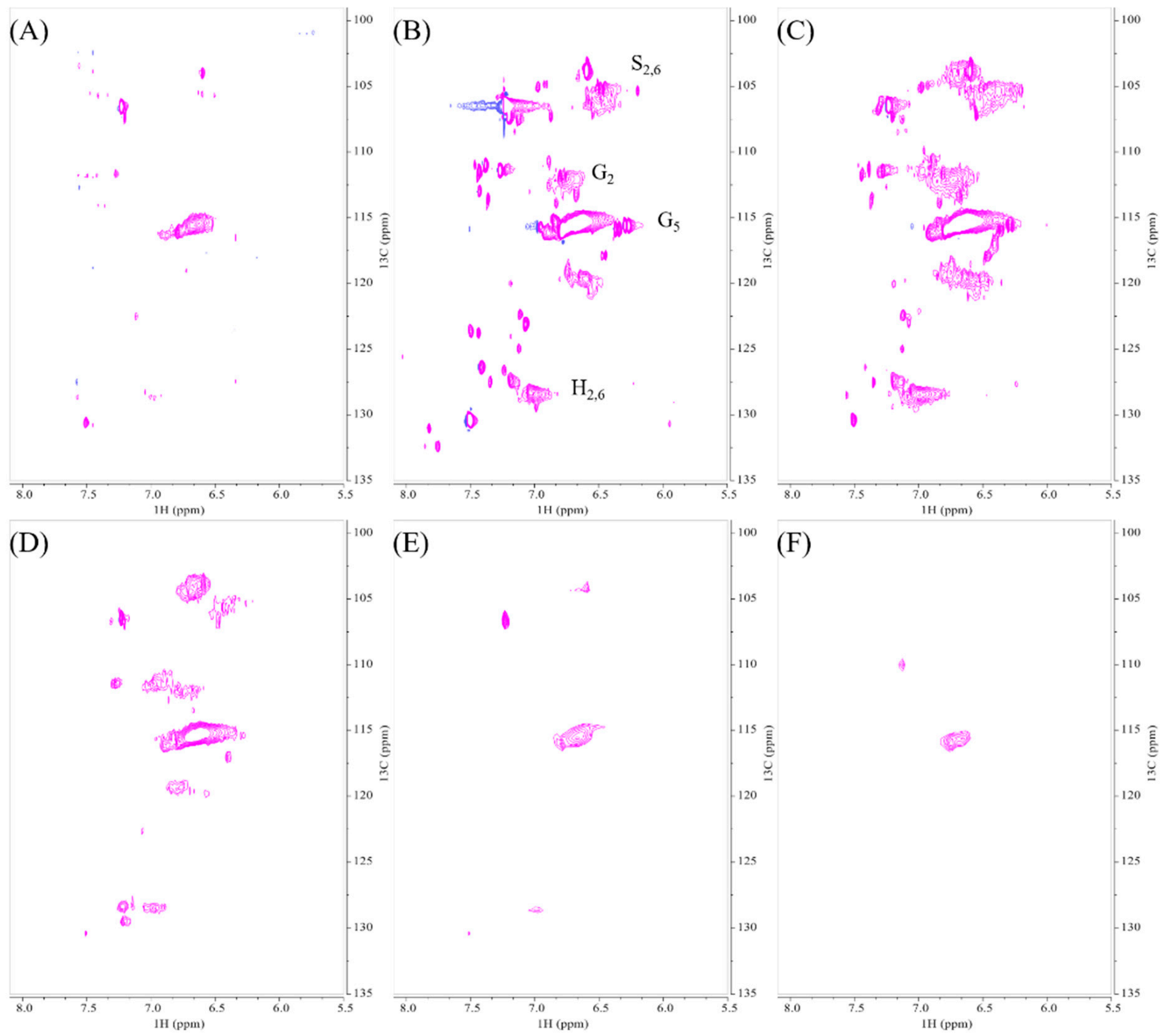

Figure S4. Sidechain regions in the 2D-HSQC NMR spectra of (A) AL, (B) FAL1, (C) FAL2, (D) FAL3, (E) FAL4, and (F) FAL5; Sn, Cn–Hn in syringyl units; Gn, Cn–Hn in guaiacyl units; Hn, Cn–Hn in hydroxyphenyl units.

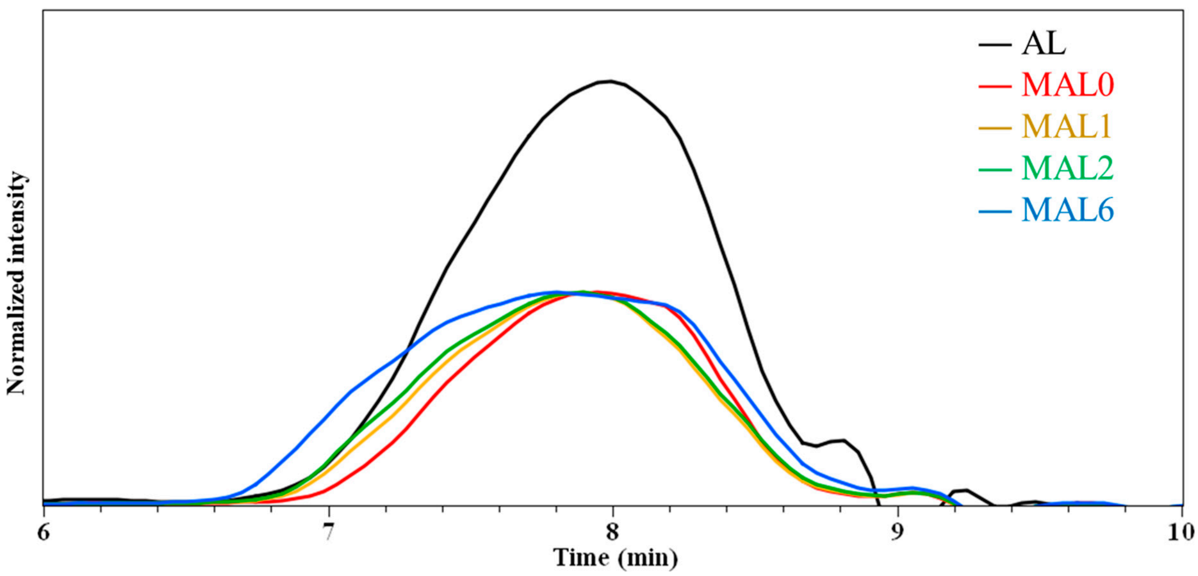

Figure S5. GPC curves of raw and methylated ALs

Table S1. Chemical and thermal properties of wheat straw soda lignin (AL)

Properties	Value
Elemental analysis (wt %)	
Carbon	60.7 ± 0.1
Hydrogen	5.9 ± 0.4
Nitrogen	0.0 ± 0.0
Oxygen*	33.4 ± 0.3
Sulfur	
Proximate analysis (wt %, dry basis)	8.6 ± 1.6
Carbohydrates*	81.6 ± 1.6
Acid-insoluble lignin	4.8 ± 0.2
Acid-soluble lignin	5.1 ± 0.1
Ash	60.7 ± 0.1

* calculated by difference

Table S2. Solubility parameters and related properties of five different solvents used in this study

	Solvent (at 25 °C)				
	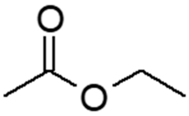 Ethyl acetate	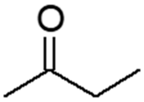 2-Butanone	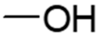 Methanol	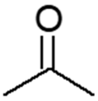 Acetone	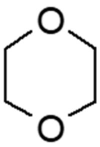 1,4-Dioxane
Hildebrand solubility parameters[a]	9.1	9.3	14.3	9.8	10.0
Hansen solubility parameters[b]	18.1	19.1	29.6	20.0	20.5
Polar component[b]	5.3	9.0	12.3	10.4	1.8
Hydrogen bonding component[b]	7.2	5.1	22.3	7.0	7.4

[a] Hansen (1967)

[b] Barton (1991)

Table S3. Changing the content of phenolic hydroxyl groups in methylated AL quantified by 31P NMR and their GPC information

	Phenolic hydroxyl (mmol g^{-1})				Carboxylic acids (mmol g^{-1})	Aliphatic (mmol g^{-1})	M_w	M_n	M_w/M_n
	H	G	S	4-O-5					
AL	0.29	0.91	0.81	0.14	1.31	2.38	2,880	1,130	2.6
MAL0	0.22	0.87	0.78	0.14	1.25	2.22	2,696	1,248	2.2
MAL1	0.15	0.55	0.43	0.05	1.18	2.30	3,291	1,372	2.4
MAL2	0.08	0.30	0.20	0.03	0.61	2.35	3,460	1,354	2.6
MAL6	ND*	0.08	0.08	0.01	0.15	1.73	4,243	1,339	3.2

* not detected

References

Barton, A.F. 1991. *CRC handbook of solubility parameters and other cohesion parameters*. CRC press.

Hansen, C.M. 1967. The three dimensional solubility parameter. *Danish Technical: Copenhagen*, **14**.
